# A novel CRISPR/Cas9 associated technology for sequence-specific nucleic acid enrichment

**DOI:** 10.1371/journal.pone.0215441

**Published:** 2019-04-18

**Authors:** Richard C. Stevens, Jennifer L. Steele, William R. Glover, Jorge F. Sanchez-Garcia, Stephen D. Simpson, Devon O’Rourke, Jordan S. Ramsdell, Matthew D. MacManes, W. Kelley Thomas, Anthony P. Shuber

**Affiliations:** 1 Genetics Research LLC, Wakefield, Massachusetts, United States of America; 2 Hubbard Center for Genome Studies, University of New Hampshire, Durham, New Hampshire, United States of America; 3 Department of Molecular Cellular and Developmental Biology, University of New Hampshire, Durham, New Hampshire, United States of America; University of Helsinki, FINLAND

## Abstract

Massively parallel sequencing technologies have made it possible to generate large quantities of sequence data. However, as research-associated information is transferred into clinical practice, cost and throughput constraints generally require sequence-specific targeted analyses. Therefore, sample enrichment methods have been developed to meet the needs of clinical sequencing applications. However, current amplification and hybrid capture enrichment methods are limited in the contiguous length of sequences for which they are able to enrich. PCR based amplification also loses methylation data and other native DNA features. We have developed a novel technology (Negative Enrichment) where we demonstrate targeting long (>10 kb) genomic regions of interest. We use the specificity of CRISPR-Cas9 single guide RNA (Cas9/sgRNA) complexes to define 5′ and 3′ termini of sequence-specific loci in genomic DNA, targeting 10 to 36 kb regions. The complexes were found to provide protection from exonucleases, by protecting the targeted sequences from degradation, resulting in enriched, double-strand, non-amplified target sequences suitable for next-generation sequencing library preparation or other downstream analyses.

## Introduction

Direct sequencing of specific loci or sets of loci can provide significant diagnostic value without sequencing the entire genome [1]. While whole genome sequencing and whole exome sequencing can generate more comprehensive assays of genetic variants, targeted sequencing has additional advantages [2]. These include a significant reduction in the technical diversity of sequences to be analyzed, reduced cost per assay, and avoidance of the ethical complications associated with the generation of sequence variants of unknown clinical significance.

Existing methods for targeted enrichment [3–6] generally involve either amplification or hybrid capture probe-based methods that utilize overlapping, tiled single strand oligonucleotides for positive enrichment to capture the desired DNA fragments. Amplification-based approaches provide highly-focused targeted sequencing, but they suffer from potential allelic bias and result in non-native DNA copied *in vitro* that lack epigenetic information. Both of these methods require optimization of annealing conditions that become more difficult as target complexity increases, limiting the number of targets that can be multiplexed. Amplification-based approaches are also typically limited to short fragments and require inference of haplotypes.

In addition to short fragments of interest, research has demonstrated that long DNA is also an important target for enrichment [7]. Long-fragment DNA sequencing allows for identification of insertions and deletions, as well as characterization of repetitive elements and gene duplications [8]. Many of the current sample preparation methods associated with long DNA enrichment result in a homogeneous sized population of DNA without sequence specific identity and therefore may not be directly applicable to clinical diagnostic applications. The CATCH method uses Cas9 to cut and extract target regions via gel purification which results in very long loci enriched directly from lysed cells [9]. Enrichment by hybridization is also used for capturing long DNA fragments from microbes, but require a complex mixture of bait sequences to achieve sufficient efficiency of capture [10].

We have developed a Negative Enrichment technology with the goal of increasing enrichment specificity, lowering cost, and increasing throughput. This CRISPR-based (Clustered Regularly Interspaced Short Palindromic Repeat) technology does not require specific targeting of background DNA and is based on the protection of specific loci from exonuclease digestion. We use Cas9/sgRNA complexes to flank target specific sequences based on chosen guide RNAs [11]. Previous observations have reported long residence times of Cas9/sgRNA on-target DNA [12, 13]. During exonuclease treatment, DNA outside the specific target loci is depleted while targeted regions are protected from exonuclease digestion by steric inhibition from the Cas9/sgRNA complexes. Because the Cas9/sgRNA complex does not exhibit the sequence-specific bias that hybridization-based approaches encounter, Negative Enrichment is suitable for a high level of multiplexing. Given that our approach does not rely on *in vitro* replication or DNA fragmentation, the technique is capable of enriching long, native DNA segments that can be coupled with a variety of approaches, including quantitative polymerase chain reaction (qPCR) and next-generation sequencing (NGS).

## Materials and methods

### Negative Enrichment for protection of DNA loci

The sgRNAs used are listed in S1 Table. Two sgRNAs representing loci separated by 10 to 36 kb were selected and bound to Cas9 Nuclease (New England Biolabs (NEB), cat. #M0386M) for 30 minutes at 25°C. The Cas9/sgRNA complexes were then mixed with target DNA and incubated for 60 minutes at 37°C. Next, exonuclease III (NEB, cat. #M0206L) and exonuclease VII (NEB, cat. #M0379S) were added with NEB buffer 1 (NEB, cat. #B7001S) and incubated for a total of 240 minutes at 37°C. Exonuclease III is a 3’ to 5’ exonuclease that acts primarily on double-stranded DNA while exonuclease VII acts on single-stranded DNA in both directions. Experiments used 330 ng starting lambda genomic DNA (NEB, cat. #N3011L) or 500 ng starting human genomic DNA (Promega, cat. #G3041). In some lambda Negative Enrichment experiments, human genomic DNA partially digested with Sau3AI was included to increase the complexity of background. For multiplex Negative Enrichment, four sgRNAs (S1 Table) were used to protect two different loci on chromosomes 7 and 17.

Agarose gel analysis was performed on an aliquot of each reaction on a 0.7% LE agarose gel (Lonza, cat. #50004) in TAE (Boston BioProducts, cat. #BM-250) for 2–3 hours at 96 volts on a Gibco-BRL Gel Electrophoresis Apparatus (cat. #21087–010) with GelRed (Biotium, cat. #41003) and imaged with a Nikon D3000 digital camera using a Stratagene 2020E Transilluminator.

Samples were phenol-chloroform extracted and ethanol precipitated using standard techniques. Samples were resuspended in 10 mM Tris, pH 7.5.

### qPCR analysis

Protected and unprotected DNA regions were quantified using FAM-based probes (IDT, S2 Table) following the manufacturer’s instructions. The qPCR analyses were performed using a Rotor-Gene Q (Qiagen, cat. #9001550) and the QuantiNova Probe PCR Kit (Qiagen, cat. #208252) following the manufacturer’s instructions. Protection was quantified by dividing the qPCR signal of protected regions by the signal of unprotected regions.

### Generation of DNA libraries and Illumina sequencing

Illumina sequencing libraries were prepared using the Kapa HyperPlus Kit (Kapa Biosystems, cat. #KK8514) following the manufacturer’s instructions. Libraries were sequenced on a HiSeq 2500 system (Illumina, Inc) with all samples run as paired-end 250 bp reads. Reads were mapped to the respective target genomes (lambda accession number NC_001416.1, with additional mutations: 37589 C→T, 45352 G→A, 37742 C→T, and 43082 G→A and human build GRCh38/hg38) using BWA (Burrows-Wheeler Aligner, version 0.7.12-r1039) [14]. For human genomic samples, reads were mapped to the GRCh38 human genome reference assembly, using BWA-MEM. The alignments were sorted and indexed using samtools (version 1.3.1) [15]. Coverage graphs were generated for each of the subsets using BEDTools (version 2.27.1) [16], which were used to create coverage plots using the UCSC Genome Browser [17].

### Generation of DNA libraries and Nanopore sequencing

Libraries for Oxford Nanopore Technologies (ONT) sequencing were prepared using the default 1D Ligation protocol provided by ONT (Kit number LSK-108). This protocol, in brief, polishes and polyadenylates the ends of DNA fragments followed by the ligation of proprietary ONT adapters. The ONT library was then loaded onto a MinION flowcell (R9.4) and sequenced for 48 hours. Raw data were basecalled using Albacore (version 2.3.1), and aligned to the reference genome using minimap2 (version 2.10) [18]. The reference consisted of the GRCh38 human genome assembly concatenated to the Lambda NEB genomic reference. The alignments were sorted and indexed using samtools (version 1.3.1), filtered based on quality, and partitioned into subsets using Pysam (version 0.13; https://github.com/pysam-developers/pysam). Coverage graphs were generated for each of the subsets using BEDTools (version 2.27.1) [16], which were used to create coverage plots using the UCSC Genome Browser [17].

## Results

### Negative Enrichment of targeted lambda DNA fragments

**Fig 1:**
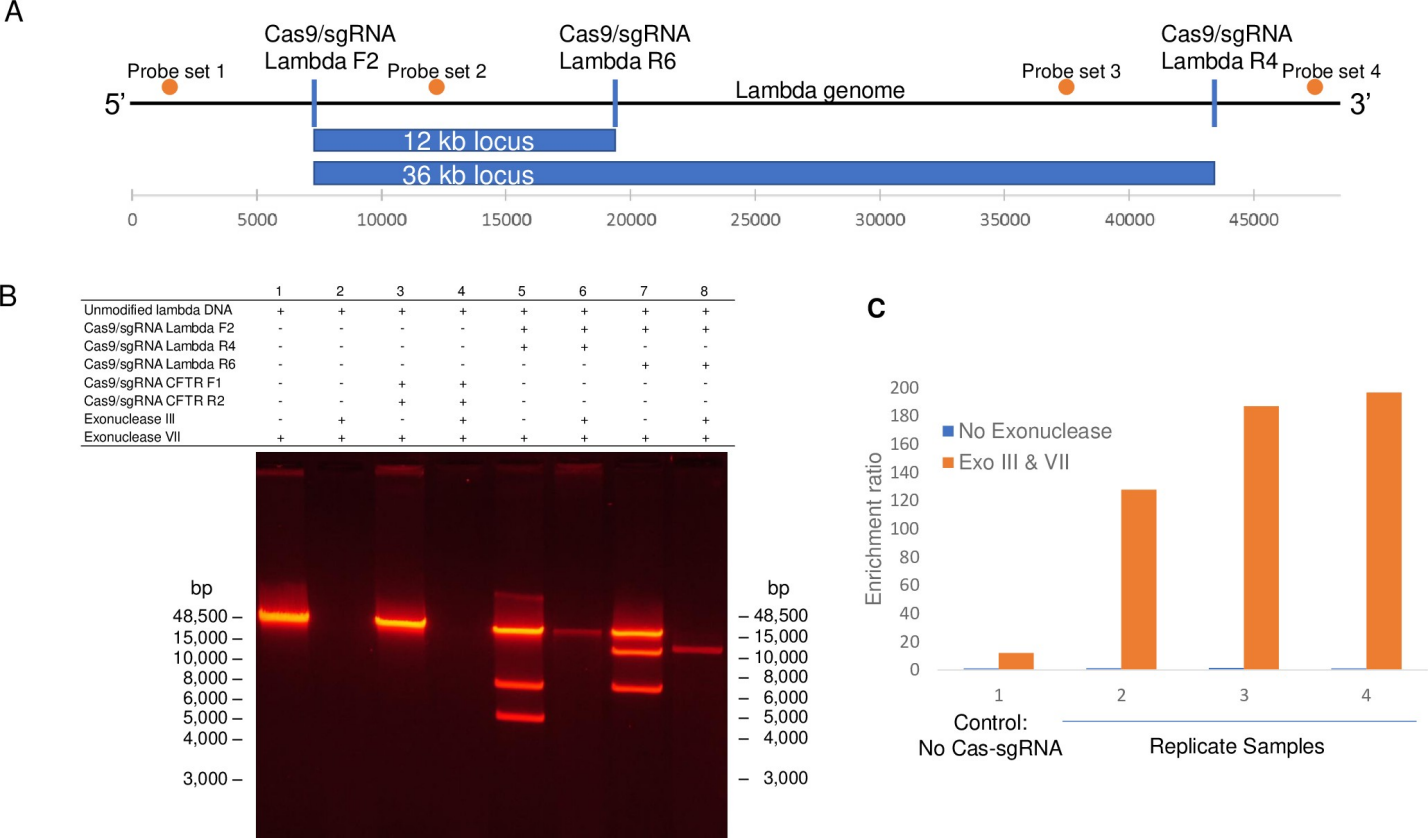
Cas9/sgRNAs protect lambda DNA from exonuclease digestion. (A) Diagram of lambda genomic DNA shows the relative positions of three Cas9/sgRNA complexes (Lambda F2, Lambda R6, and Lambda R4) and the 12 kb fragment protected by Lambda F2 and Lambda R6 or the 36 kb fragment protected by Lambda F2 and Lambda R4. Probe sets 1–4 are used in qPCR assays to determine relative enrichment of protected regions. (B) Gel electrophoresis shows lambda DNA without or with exonuclease III treatment (lanes 1 and 2); lambda DNA treated with a pair of Cas9/sgRNAs with no homology to lambda (sgRNAs CFTR F1 and CFTR R2 target human loci) with exonuclease VII (lanes 3) or with exonucleases III and VII (lane 4); lambda DNA complexed with Lambda F2 and Lambda R4 with exonuclease VII (lane 5) or with exonucleases III and VII (lanes 6); and lambda DNA complexed with Lambda F2 and Lambda R6 with exonuclease III (lane 7) or with exonucleases III and VII (lanes 8). (C) Concentrations, as determined by qPCR, of Probe set 2 (within the protected locus) were compared with the concentrations of Probe set 4 (outside of the protected locus) and presented as ratios.

Cas9/sgRNA complexes were designed to flank two overlapping regions of the lambda genome (Fig 1A). The 5′ end of the region of interest was defined by a single Cas9/sgRNA complex (referred to as Lambda F2) with the 3’ ends defined by two additional guide RNA complexes: Lambda R6 (12 kb from F2) and Lambda R4 (36 kb from F2). Lambda F2 and Lambda R6 Cas9/sgRNAs complexes generate three fragments (7, 12, 29 kb) with the 12 kb fragment protected from exonuclease digestion. Combination of the Lambda F2 and Lambda R4 Cas9/sgRNAs complexes generate three fragments (7, 36, 5 kb) with the 36 kb fragment protected from exonuclease digestion.

The binding and protection of Lambda F2, Lambda R6, and Lambda R4 Cas9/sgRNA complexes from exonuclease digestion is demonstrated in Fig 1B. Exonuclease controls (Lane 1 and 2) and sequence specific guide RNA controls (Lane 3 and 4) are shown in Fig 1B. Lambda genomic DNA was incubated with Lambda F2 and R4 Cas9/sgRNA complexes yielding fragments of the 5, 7, and 36 kb sizes (lane 5). Exonuclease sample treatment resulted in the protected 36 kb fragment (lane 6). In addition, the Lambda F2 and R6 Cas9/sgRNA complexes cleaved the target sequence to result in 7, 12, and 29 kb fragments (lane 7). Exonuclease treatment with Cas9/sgRNA protected the 12 kb fragment while the adjacent 7 and 29 kb fragments were digested (lane 8).

The level of enrichment and specificity of the targeted sequences in the presence of partially digested human DNA were determined by qPCR analyses of the protected and unprotected regions after exonuclease digestions (Fig 1 and S2 Table). The enrichment ratios were calculated by dividing the qPCR value for an on target site (Probe set 2) by the qPCR value for an off target site (Probe set 4). In the absence of exonuclease with and without Cas9/sgRNA complexes, the ratios of template concentration based on the internal Probe set 2 to the external Probe sets 1, 3, 4 were consistent with equimolar concentrations inside and outside of the protected region (Fig 1C). The control sample with no Cas9/sgRNA protection and treatment with exonuclease had ratios greater than the expected 1:1 suggesting variability in exonuclease efficiency. Inter-sample enrichment ratios of 127x to 197x were observed when Probes 2 and 4 were compared (Fig 1C).

**Fig 2:**
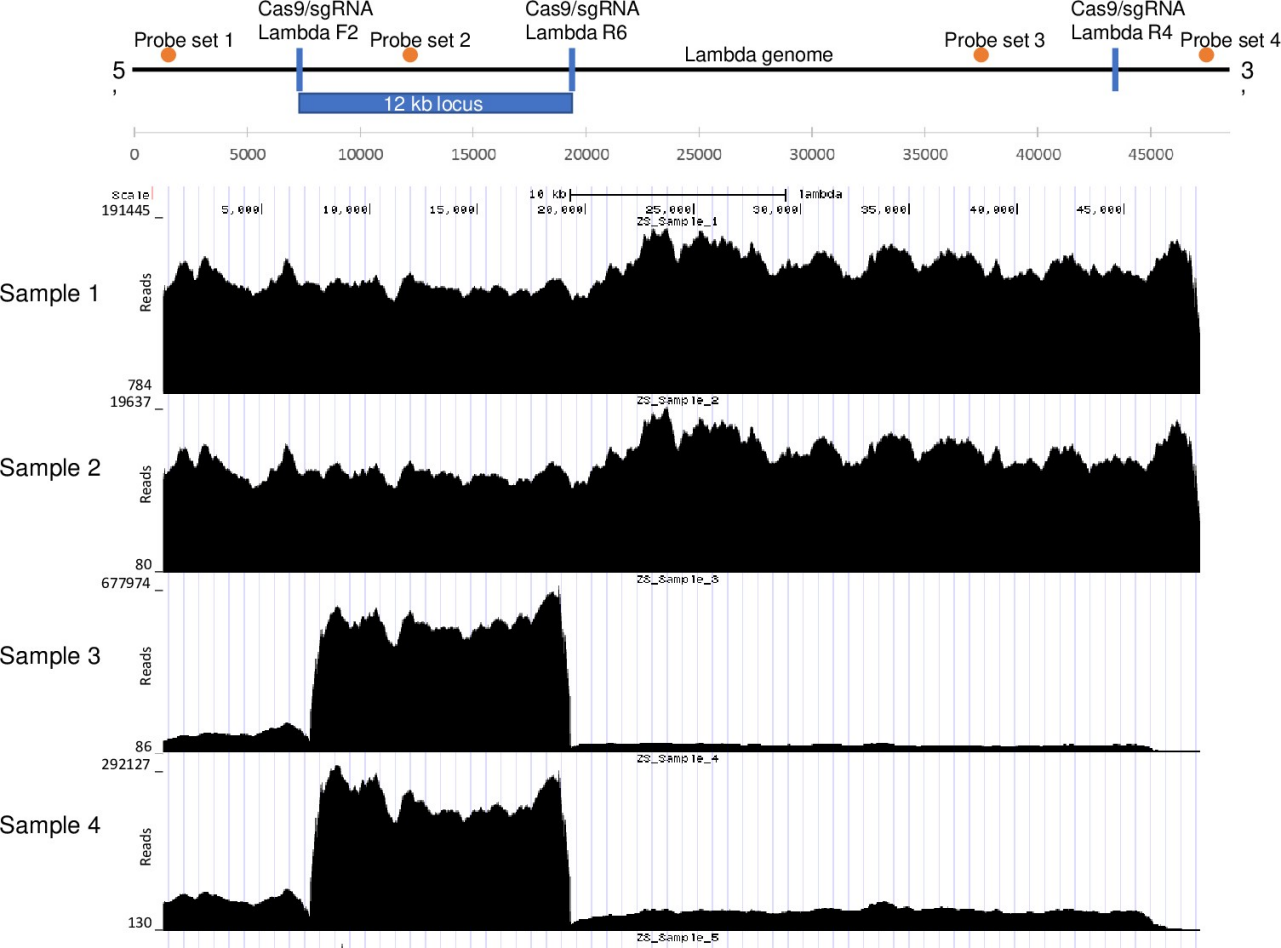
Illumina sequence analyses demonstrate enrichment of the protected 12 kb lambda DNA region. Plots of aligned Illumina reads of the protection experiment mapped to the lambda target genome using BWA 0.7.12-r1039. Sample 1 had no Cas9/sgRNA and no exonuclease treatment with a 120,000:1 copy ratio of lambda to human DNA. Sample 2 had no Cas9/sgRNA and no exonuclease treatment with a 3,000:1 copy ratio of lambda to human DNA. Sample 3 had Cas9/sgRNA Lambda F2 and Lambda R6 treatment with exonucleases III and VII with a 120,000:1 lambda DNA to human genomic DNA copy ratio. Sample 4 had Cas9/sgRNA Lambda F2 and Lambda R6 treatment with exonucleases III and VII with a 3000:1 lambda DNA to human genomic DNA copy ratio.

In addition to gel electrophoresis and qPCR analysis, NGS Illumina sequencing was used to further validate the 12 kb fragment protection within lambda in a background of human genomic DNA at copy ratios of 120,000:1 and 3,000:1 lambda to human genomic DNA. Sequencing results yielded approximately 7417 Mb of data (120,000:1 ratio) and 4636 Mb of data (3,000:1 ratio), contained in 30 million and 19 million sequencing reads, respectively. As shown in Fig 2, the reads mapped in the two protected and exonuclease treated samples demonstrate a significant and well-defined enrichment of the protected fragments (samples 3 and 4). Visual inspection of the sequence plots in Fig 2 shows that the coverage declined sharply at the termini of the enriched fragments.

In order to determine if the 12 kb lambda enrichment consisted of contiguous and intact full-length fragments, analysis was performed on a long read sequencing platform (Oxford Nanopore MinION). Sequencing results yielded approximately 770 Mb of data, contained in 124,833 sequencing reads, each of which correspond to a single sequenced molecule. The N50 of these reads was 10,713 bp, with the longest read being 105,551 bp.

**Fig 3:**
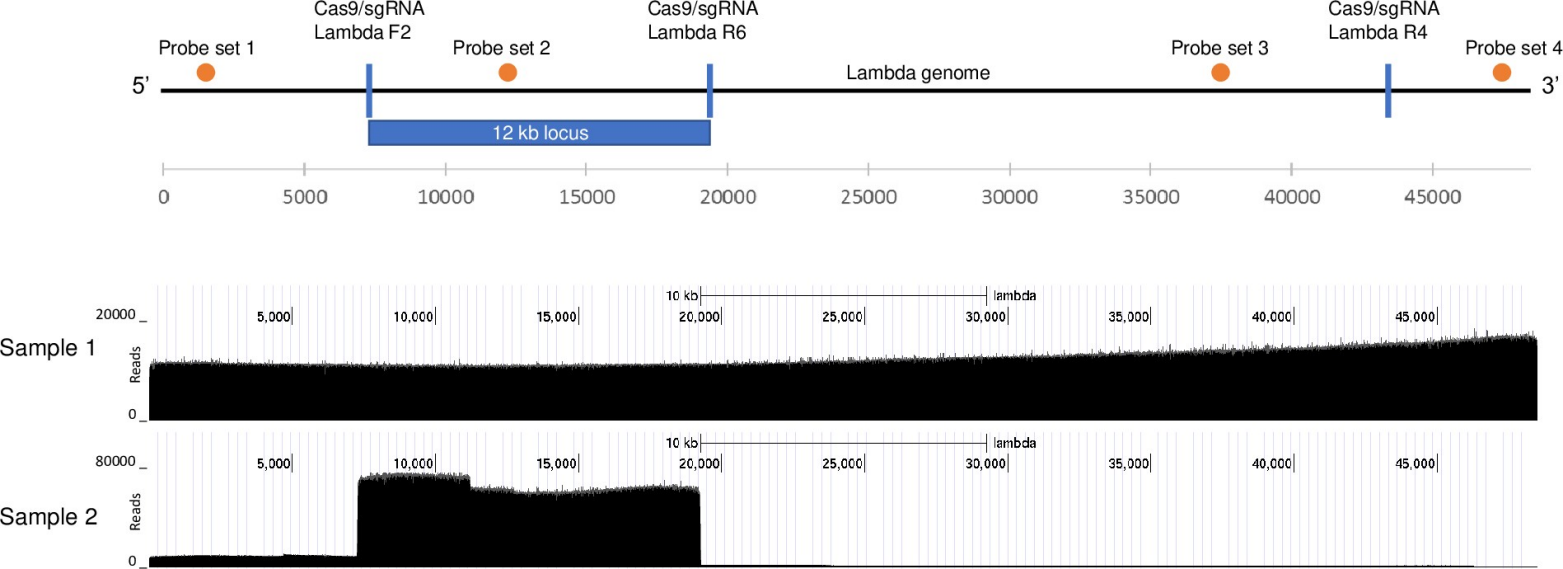
Nanopore analyses demonstrate enrichment of the protected 12 kb lambda DNA region. Plots of aligned Nanopore sequencing results for lambda Negative Enrichment. Sample 1 had lambda DNA without exonuclease. Sample 2 had lambda DNA treated with Lambda F2 and Lambda R6 Cas-RNA complexes and exonucleases III and VII.

Following data processing of the 12 kb lambda protection (120,000:1 copy ratio), the majority of reads (98%) mapped to lambda with enrichment observed between coordinates 7,269 to 19,264 bp of the lambda genome representing 88.8% of the coverage is in the protected locus and an associated enrichment ratio of 23.9 (Fig 3). A significant fraction of the reads spans the full target region. In addition, when only molecules that are between 11kb and 13 kb in size are considered, 98.8% of the coverage is located in the protected locus, with an enrichment ratio of 242.0.

Endonuclease-deficient Cas9 (dCas9) was also used to determine if protection could occur in the absence of cleavage. Initial experiments indicate that dCas9 is similarly effective as Cas9 in the protection of DNA loci (S1 Fig).

### Negative Enrichment of human genomic targets

**Fig 4:**
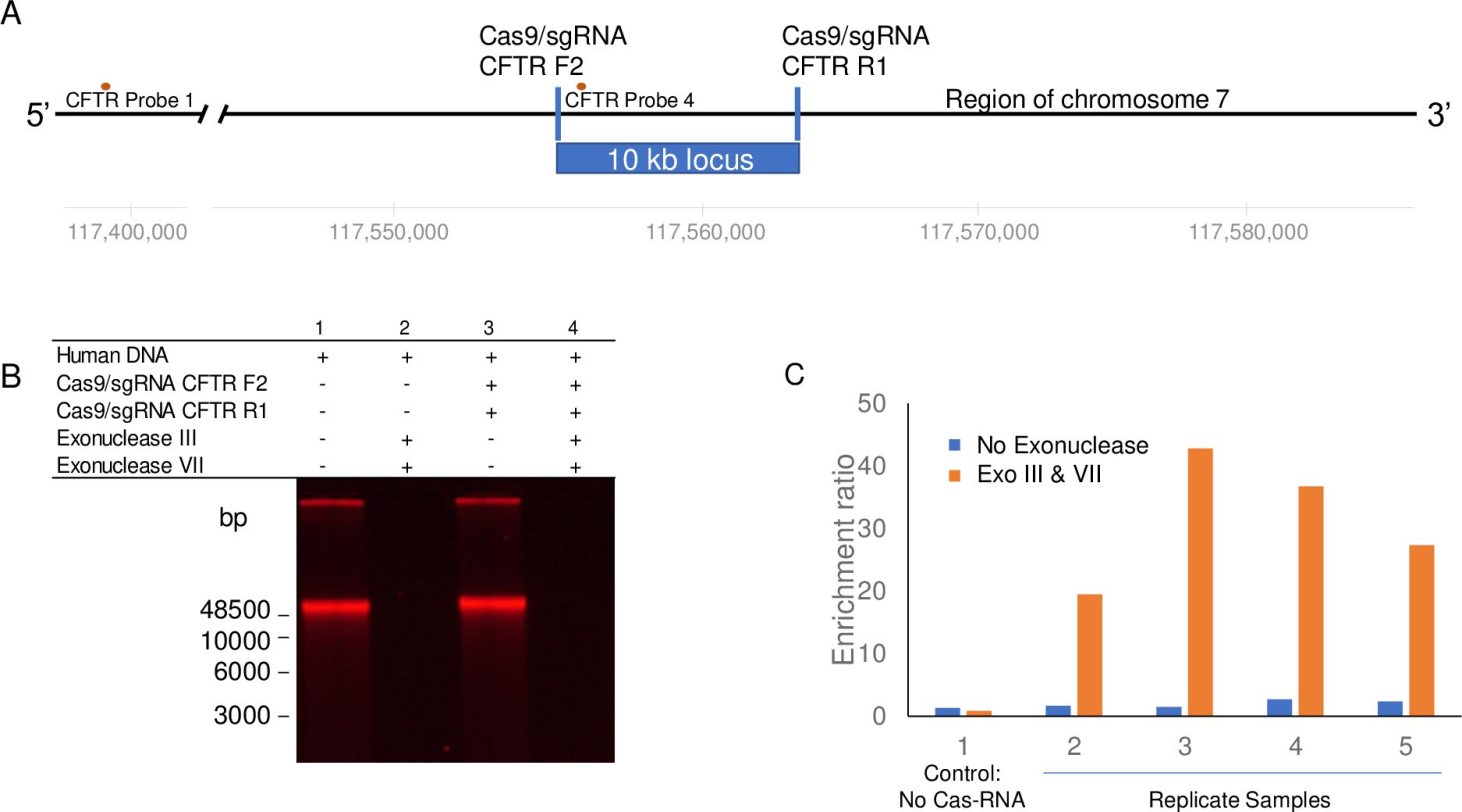
Cas9/sgRNAs protect a target 10 kb human CFTR gene sequence from exonuclease digestion. (A) Chromosome 7 map showing the location of the cystic fibrosis transmembrane conductance regulator (CFTR) gene, the relative position of the Cas9/sgRNA complexes that bracket the 10 kb target fragment, and the two qPCR probes (CFTR Probe 1, CFTR Probe 4). (B) Bar graph of qPCR analyses using the internal and external probe data comparing samples without and with exonuclease treatment. Sample 1 is no Cas9/sgRNA treatment. Samples 2–5 are replicate samples of Cas9/sgRNA treatment.

Negative Enrichment was also applied to human genomic DNA. We designed Cas9/sgRNA complexes flanking a 10 kb CFTR gene sequence (Fig 4A). As we observed with lambda DNA, exonucleases efficiently digested non-targeted high molecular weight human genomic DNA as demonstrated in S2 Fig lane 2. Quantification of protection was performed by qPCR due to the relative low abundance of single copy targeted sequence in the human genome (S2 Fig lane 4).

Analysis by qPCR was conducted using one probe set internal to the 10 kb CFTR region and an external probe 151 kb distant from the target (Fig 4A). The enrichment ratios were calculated by dividing the qPCR value for an on target site (Probe set 4) by the qPCR value for an off target site (Probe set 1 Consistent enrichment of the protected region was observed (Fig 4B and Table 1). As shown in Table 1, samples untreated with Cas9/sgRNAs (samples 1a and 1b) demonstrate uniform exonuclease digestion of internal and external regions. Samples 2 through 5 are replicate samples of treatment with the CFTR F2 and CFTR R1 Cas9/sgRNAs without (samples 2a-5a) and with exonuclease treatment (2b-5b).

**Table 1:** qPCR analyses of Cas9/sgRNA Negative Enrichment protection of the target CFTR DNA fragment.

Sample	Cas9-sgRNAs	Exonuclease III/VII	qPCR Probe 4 / 1 ratio (enrichment)
1a	-	-	1.37
1b	-	+	0.85
2a	+	-	1.67
3a	+	-	1.5
4a	+	-	2.73
5a	+	-	2.37
2b	+	+	19.49
3b	+	+	42.82
4b	+	+	36.76
5b	+	+	27.35

**Fig 5:**
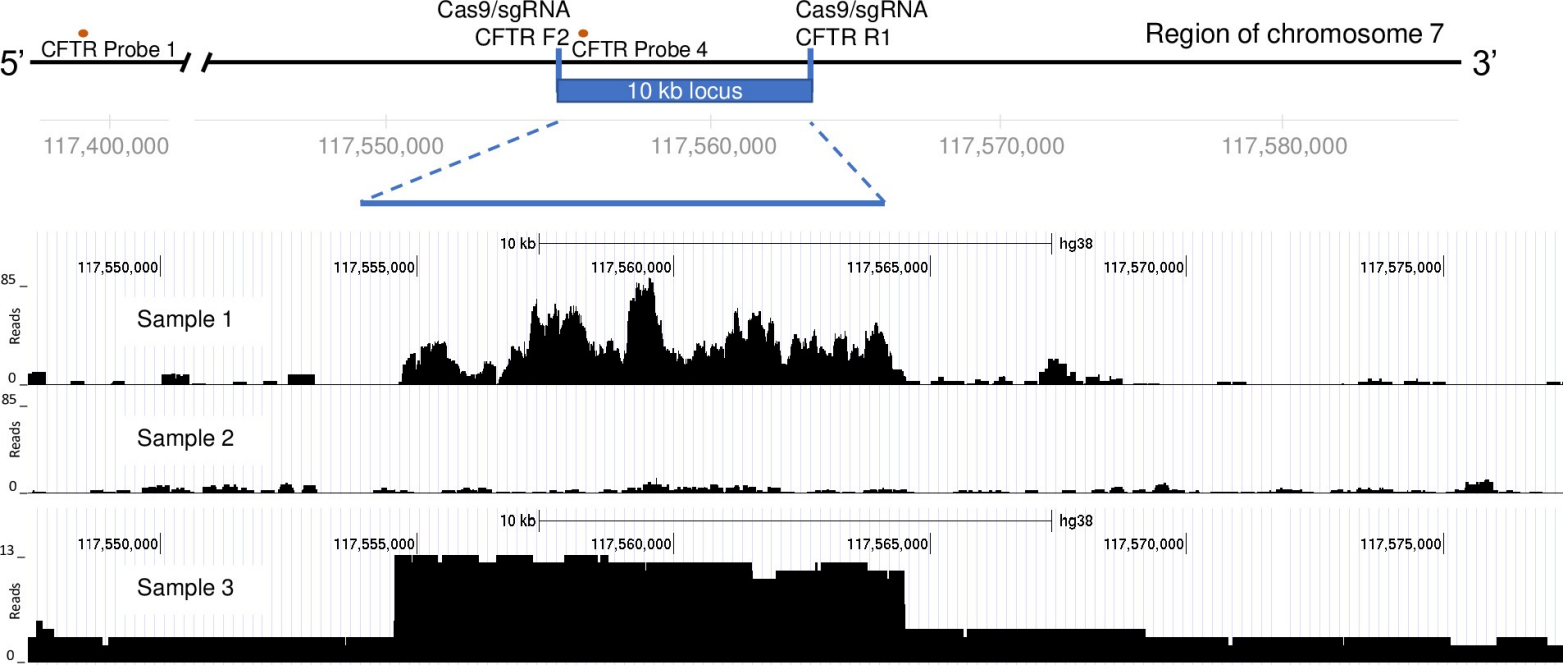
Illumina and Nanopore sequencing analyses demonstrate enrichment of the target human CFTR genomic protected region. NGS reads mapping to the CFTR region from an Illumina short read library prepared from Cas9/sgRNA and exonuclease treated human genomic DNA (Sample 1) and a control sample with no Cas9/sgRNA nor exonuclease treatment (Sample 2). Sample 3 is the plot of long-read Nanopore sequencing from a library prepared from Cas9/sgRNA and exonuclease treated human genomic DNA.

Illumina short-read sequencing was then performed on the samples described in Table 1 and mapped against the reference human genome. As shown in Fig 5, reads from the protected and exonuclease treated samples demonstrated 20x to 43x enrichment ratio enrichment when compared with the average of four unenriched genomes. Nanopore sequencing results of enriched fragments confirmed that the enriched 10 kb target primarily consisted of contiguous fragments (10/13 mapped reads).

### Negative Enrichment of multiplexed human genomic targets

**Fig 6:**
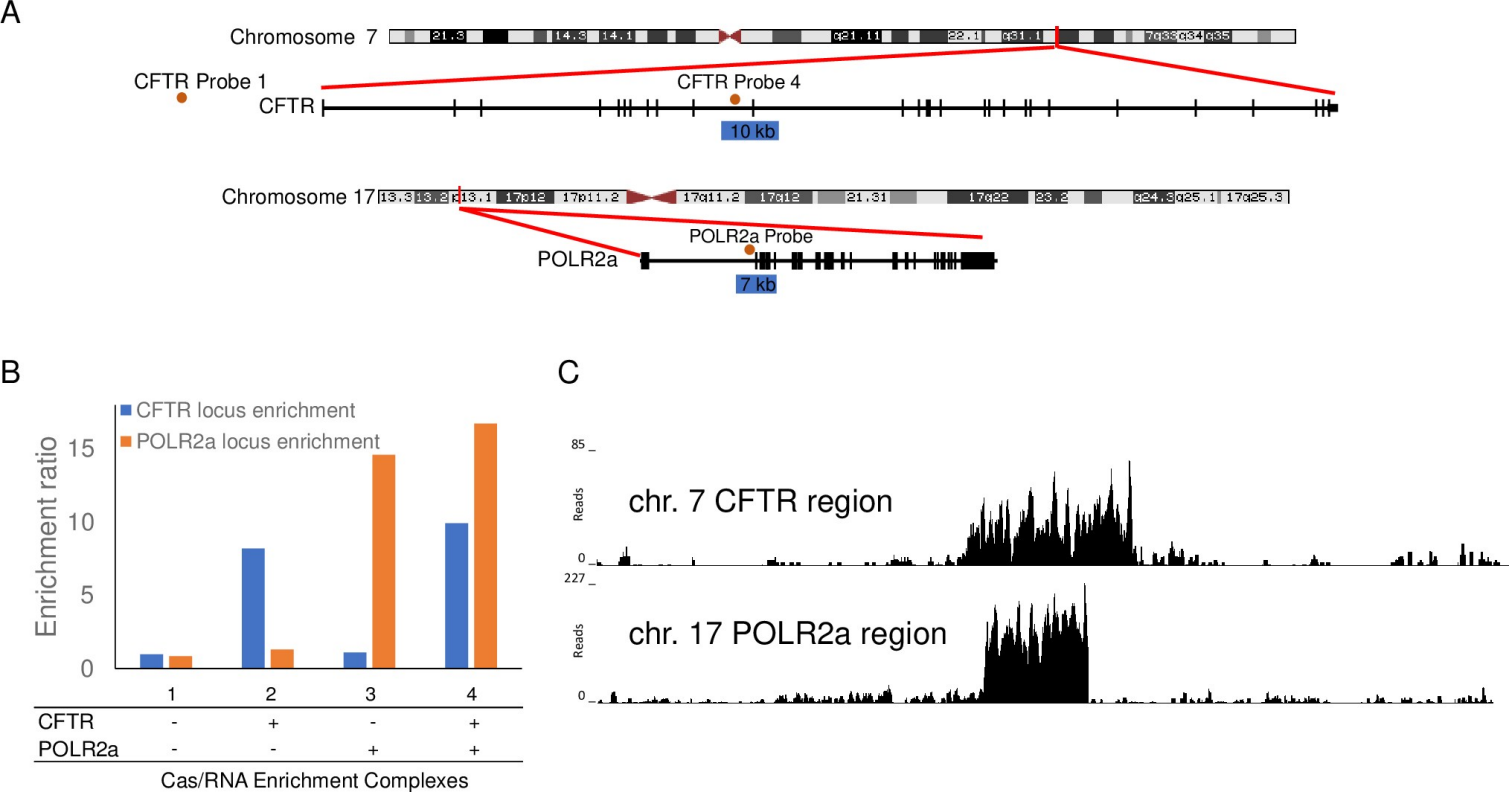
Targets for Cas9/sgRNA multiplexing protection experiments. (A) Diagram showing the position of two Cas9/sgRNA complexes within the CFTR gene on human chromosome 7, two Cas9/sgRNA complexes within the POLR2a gene on human chromosome 17, and the location of the qPCR probes used in the analyses. (B) qPCR analyses demonstrating multiplexing Cas9/sgRNA protection of human genomic targets. Ratios of on target and off target results for exonuclease treated and non-exonuclease treated samples. Sample 1, no protection; Sample 2, CFTR protection; Sample 3, POLR2a protection; Sample 4, average of four independently performed CFTR/POLR2a multiplex protection reactions. (C) NGS reads mapping to two regions from an NGS library prepared from Cas9/sgRNA and exonuclease treated human genomic DNA for both CFTR 10 kb protection and POLR2a 7 kb protection.

Multiplexed Cas9/sgRNA complexes were used to target two different human genomic loci: the 10 kb CFTR locus on chromosome 7 and a 7 kb locus in the polymerase RNA II (DNA directed) polypeptide (POLR2a) gene on chromosome 17 (Fig 6). We quantified the Cas9/sgRNA protection using the POLR2a Probe set and CFTR Probe set 4 listed in S2 Table. The qPCR data shown in Fig 6B demonstrates Cas9/sgRNA protection of the two different chromosomal loci performed separately (samples 2–3) and combined (sample 4). The independent and multiplex enrichment of the 10 kb CFTR loci were similar (8.2 vs. 9.9). Enrichment of the POLR2a target sequence was 14.6x and16.7x enrichment ratio respectively when performed as an independent and multiplex enrichment. We present the data as normalized against the no exonuclease controls to allow for comparisons even when different loci exhibit different sensitivities to library preparation and exonuclease sensitivity. Independently to the qPCR results, sequencing results also demonstrate enrichment of the two targeted regions in the multiplex reaction (Fig 6B and 6C).

## Discussion

Targeted enrichment [3–6] generally involves either amplification or hybrid capture probe-based methods that include overlapping, tiled baits for positive enrichment of targeted sequences. Amplification-based approaches provide highly-focused targeted sequencing, but they suffer from potential allelic bias and result in non-native DNA sequence. We have developed a novel sequence specific enrichment methodology that results in enriched native DNA sequence and does not require target amplification or target denaturation and hybridization.

Here we demonstrate Negative Enrichment of 10 to 36 kb regions in simple (lambda) and complex (human) genomes using a CRISPR-based Cas9/sgRNA system where the targeting and purification of target regions retains biological information and allows for long fragments to be collected. While other long DNA enrichment methods require complex instrumentation and detailed knowledge of off-target DNA, this method is simple, robust and requires only information about the target sites to be enriched.

Successful enrichment was demonstrated here with little optimization, demonstrating the simplicity and ease of application. In addition, this approach can be applied with simple modifications addressing the presence of nicked DNA which present significant difficulty for amplification and hybrid capture of long targets. We have also explored the use of different Cas variants (e.g. dCas9, Cas12a) for similar Negative Enrichment approaches. The Supplemental Information demonstrates preliminary evidence for Cas12a Negative Enrichment (S4 and S5 Figs).

In addition to the current long DNA targets presented here, application of this novel approach may also be applied to the protection and enrichment of short DNA targets. The ability to characterize small DNA targets using Negative Enrichment will be useful in any area of research or medicine where haplotype or polymorphisms need to be characterized. We are currently exploring methods to use Negative Enrichment on small targets including the 180 bp fragments found in human blood as well as FFPE associated DNA. Characterizing circulating-free DNA (cfDNA) is an area of active research in many laboratories and could have significant clinical importance. We are also investigating single base discrimination by designing Cas9/sgRNA complexes with specific base mismatches to target DNA and applying Negative Enrichment. Additionally, we have observed that Cas9/sgRNA complexes are active in neat plasma. Enrichment of cfDNA eliminating the need for standard DNA preparations prior to enrichment would enable current research and clinical investigations and direct positive enrichment from human plasma using Cas9 is an ongoing effort in our laboratory.

Most targeted capture and resequencing methods rely on short read libraries that eliminate biological information or create allelic bias due to amplification before the capture process. Here, we demonstrate Negative Enrichment of 10 to 36 kb regions in simple (lambda) and complex (human) genomes using a CRISPR-based Cas9/sgRNA system where the targeting and purification of target regions retains biological information and allows for long fragments to be analyzed as a single contiguous sequence. Negative Enrichment overcomes many of the limitations that exist with the current targeted sequence enrichment methodologies and is agnostic to existing analytical platforms.

## Supporting information

S1 Table Cas9/sgRNAs and Cas12a/crRNAs used in this study*. (DOCX)

S2 Table qPCR probe sets used in this study*. (DOCX)

S3 Table Economic Comparison of sequencing costs of enriched versus unenriched samples. (DOCX)

S1 Fig dCas9/sgRNA protects lambda DNA from exonuclease digestion. Gel electrophoresis of lambda DNA without or with exonuclease III and exonuclease VII treatment (lanes 1 and 2); lambda DNA with Cas9 complexed to Lambda F2 and Lambda R6 without or with exonuclease III and exonuclease VII (lane 3 and 4); lambda DNA with dCas9 complexed to Lambda F2 and Lambda R6 without or with exonuclease III and exonuclease VII (lane 5 and 6).
(TIF)

S2 Fig Agarose gel for Cas9/sgRNAs protection of a 10 kb human CFTR gene target shows complete exonuclease digestion. Gel electrophoresis showing human genomic DNA without or with exonuclease treatment (lanes 1 and 2), and human genomic DNA complexed with Cas9/sgRNAs CFTR F2 and CFTR R1 without or with exonuclease treatment (lanes 3 and 4).
(TIF)

S3 Fig Negative Enrichment using CRISPR nucleases. (TIF)

S4 Fig Exonuclease digests unprotected long lambda DNA. (A) Diagram showing intact lambda genomic DNA cos sites modified via polymerase incorporation of dGTP-αS modifications. (B) A 0.7% agarose gel shows lambda DNA filled in with wild-type dNTPs (lane 1), lambda DNA filled in with wild-type dNTPs and incubated with exonuclease III (lane 2), lambda DNA filled in with phosphorothioated bases (dGαS lambda DNA) (lane 3), dGαS lambda DNA incubated with exonuclease III (lane 4), lanes 6–9 duplicate the conditions of 1–4 with the addition of exonuclease VII.
(TIF)

S5 Fig Cas12a/crRNA followed by extension with phosphorothioated nucleotides protects lambda DNA from exonuclease digestion. (A) Diagram of lambda genomic DNA shows the relative positions of complexes Cas12a/crRNA Lambda R1 and Cas12a-crRNA Lambda F2: (B) A 0.7% agarose gel shows lambda DNA filled in with wild-type dNTPs (lane 1), lambda DNA filled in with wild-type dNTPs and incubated with exonuclease III (lane 2), lambda DNA filled in with phosphorothioated bases (dAαS lambda DNA) (lane 3), dAαS lambda DNA incubated with exonuclease III (lane 4), lambda DNA filled in with phosphorothioated bases (dGαS lambda DNA) (lane 5), dGαS lambda DNA incubated with exonuclease III (lane 6). Lambda DNA treated with Cas12a/crRNA and filled in with wild-type dNTPs (lane 7), lambda DNA filled in with wild-type dNTPs and incubated with exonuclease III (lane 8), lambda DNA filled in with phosphorothioated bases (dAαS lambda DNA) (lane 9), dAαS lambda DNA incubated with exonuclease III (lane 10), lambda DNA filled in with phosphorothioated bases (dGαS lambda DNA) (lane 11), dGαS lambda DNA incubated with exonuclease III (lane 12).
(TIF)

S1 File Alternative Negative Enrichment. (DOCX)

S2 File Cost Comparison. (DOCX)
