# Supplementary material for: A novel CRISPR/Cas9 associated technology for sequence-specific nucleic acid enrichment
Source: PLoS One. 2019 Apr 18;14(4):e0215441. doi: 10.1371/journal.pone.0215441 (PMC6472885; doi:10.1371/journal.pone.0215441)
Supplement: S1 Table — (DOCX) [file pone.0215441.s001.docx]

### S1 Table. Cas9/sgRNAs and Cas12a/crRNAs used in this study*

| **sgRNA or crRNA** | **Sequence** |
| --- | --- |
| crRNA lambda F2 | UAAUUUCUACUCUUGUAGAUAGUAUUGGUUCACACGGACA |
| crRNA lambda R1 | UAAUUUCUACUCUUGUAGAUGUCUCGUCGCUGGCAGCCUC |
| sgRNA lambda F2 | GACCGUGAUGUCAGCCUGACGGUUUUAGAGCUAGAAAUAGCAAGUUAAAAUAAGGCUAGUCCGUUAUCAACUUGAAAAAGUGGCACCGAGUCGGUGCUUUU |
| sgRNA lambda R6 | GCGCUCACAUAACGUCCACGCGUUUUAGAGCUAGAAAUAGCAAGUUAAAAUAAGGCUAGUCCGUUAUCAACUUGAAAAAGUGGCACCGAGUCGGUGCUUUU |
| sgRNA lambda R4 | GAAUCGCCCACCGAGAUAAGCGUUUUAGAGCUAGAAAUAGCAAGUUAAAAUAAGGCUAGUCCGUUAUCAACUUGAAAAAGUGGCACCGAGUCGGUGCUUUU |
| sgRNA CFTR F1 | ACAAAGCUAGUGAAUGAAUGGUUUUAGAGCUAGAAAUAGCAAGUUAAAAUAAGGCUAGUCCGUUAUCAACUUGAAAAAGUGGCACCGAGUCGGUGCUUUU |
| sgRNA CFTR R2 | UCUUAGGUCACUGAAAAUAGGUUUUAGAGCUAGAAAUAGCAAGUUAAAAUAAGGCUAGUCCGUUAUCAACUUGAAAAAGUGGCACCGAGUCGGUGCUUUU |
| sgRNA CFTR F2 | GGGGCUGGUAGUGUGAAGAUGUUUUAGAGCUAGAAAUAGCAAGUUAAAAUAAGGCUAGUCCGUUAUCAACUUGAAAAAGUGGCACCGAGUCGGUGCUUUU |
| sgRNA CFTR R1 | UUAACCAUCAAGGAUUCUGUGUUUUAGAGCUAGAAAUAGCAAGUUAAAAUAAGGCUAGUCCGUUAUCAACUUGAAAAAGUGGCACCGAGUCGGUGCUUUU |
| sgRNA POLR2a F1 | AGAGUGUAUGGAGGUACGGAGUUUUAGAGCUAGAAAUAGCAAGUUAAAAUAAGGCUAGUCCGUUAUCAACUUGAAAAAGUGGCACCGAGUCGGUGCUUUU |
| sgRNA POLR2a R6 | CAGGAAGGGCUUUCCAACGGGUUUUAGAGCUAGAAAUAGCAAGUUAAAAUAAGGCUAGUCCGUUAUCAACUUGAAAAAGUGGCACCGAGUCGGUGCUUUU |
| crRNA CF10 F2 | UAAUUUCUACUCUUGUAGAUAAGGAAGAGGGGGCUGGUAG |
| crRNA CF10 R1 | UAAUUUCUACUCUUGUAGAUGAUCAUAAGAACUUGAAAUA |

*Purchased from Integrated DNA Technologies (IDT, Skokie, Illinois)
