# Supplementary material for: A novel CRISPR/Cas9 associated technology for sequence-specific nucleic acid enrichment
Source: PLoS One. 2019 Apr 18;14(4):e0215441. doi: 10.1371/journal.pone.0215441 (PMC6472885; doi:10.1371/journal.pone.0215441)
Supplement: S2 Table — (DOCX) [file pone.0215441.s002.docx]

### S2 Table. qPCR probe sets used in this study*

| **Probe set** | **Sequences** |
| --- | --- |
| Lambda Probe set 1 | Forward: TATGGGCTGTGCACTACTGG |
|  | Reverse: TGCTGAAGCAATACCACTCG |
|  | FAM Probe: GTCCACCTGGGCTAACTCCT |
| Lambda Probe set 2 | Forward: ATTTGTGGAAGGCGGAGAG |
|  | Reverse: ACTCCAGCGTCTCATCTTTATG |
|  | FAM Probe: AGGGATTTCTCCTGTGCAGACAGC |
| Lambda Probe set 3 | Forward: CTGGCGATTGAAGGGCTAAA |
|  | Reverse: TCAGGCGTTGGTGCTTTATT |
|  | FAM Probe: TGCTTATAACGCCGCATTGCTTGC |
| Lambda Probe set 4 | Forward: CTGCATGGGTCCACTTATTT |
|  | Reverse: TGCATTCATGGCATATCTTACT |
|  | FAM Probe: ACTCACAAACTGTCTAGCCTAGAAATACCG |
| CFTR Probe set 1 | Forward: TATGGGCTGTGCACTACTGG |
|  | Reverse: TGCTGAAGCAATACCACTCG |
|  | FAM Probe: GTCCACCTGGGCTAACTCCT |
| CFTR Probe set 4 | Forward: CTGCATGGGTCCACTTATTT |
|  | Reverse: TGCATTCATGGCATATCTTACT |
|  | FAM Probe: ACTCACAAACTGTCTAGCCTAGAAATACCG |
| PolR2a  Probe set | Forward: CTGTTCAGTTGTTGGCCTTTC |
|  | Reverse: CTCAGTCGTCTCTGGGTATTTG |
|  | FAM Probe: TCCGTCACAGACATTCGCTTCTGC |

*Purchased from Integrated DNA Technologies (IDT, Skokie, Illinois)
