# Supplementary material for: A novel CRISPR/Cas9 associated technology for sequence-specific nucleic acid enrichment
Source: PLoS One. 2019 Apr 18;14(4):e0215441. doi: 10.1371/journal.pone.0215441 (PMC6472885; doi:10.1371/journal.pone.0215441)
Supplement: S3 Table — (DOCX) [file pone.0215441.s003.docx]

### S3 Table. Economic Comparison of sequencing costs of enriched versus unenriched samples

| **Characteristic** | **Parameter** | **Unenriched Sample** | **Enriched Sample** |
| --- | --- | --- | --- |
| **Reads required^a,b^** | Total (24x observed enrichment) | 185,254,390 | 7,704,974 |
|  | On target | 506 | 510 |
| **Costs^c^** | Enrichment reagents | - | $5 |
|  | Probe costs ($0.26/probe) | - | $0.52 |
|  | Library Prep: KAPA kit, $37.50/sample | $37.50 | $37.50 |
|  | Library QC: Bioanalyzer 2100, $48.87/chip | $4.44 | $4.44 |
|  | Sequencing: HiSeq (250 bp, paired ends), $3,500/lane | 49% of lane, $1,715 | 2% of lane, $70 |
|  | Total | $1,756.94 | $117.46 |
| **Time** | Enrich | - | ½ day |
|  | Library preparation | ½ day | ½ day |
|  | HiSeq | 3 days | 3 days |
|  | Total | 3 ½ days | 4 days |

^a^13x coverage, with 99.99% of bases read ≥2x.

^b^Assumes optimized cluster densities.

^c^Costs based on full lane runs, lane/pool costs shared proportionately.
