# Supplementary figures and images for: A novel CRISPR/Cas9 associated technology for sequence-specific nucleic acid enrichment
Source: PLoS One. 2019 Apr 18;14(4):e0215441. doi: 10.1371/journal.pone.0215441 (PMC6472885; doi:10.1371/journal.pone.0215441)

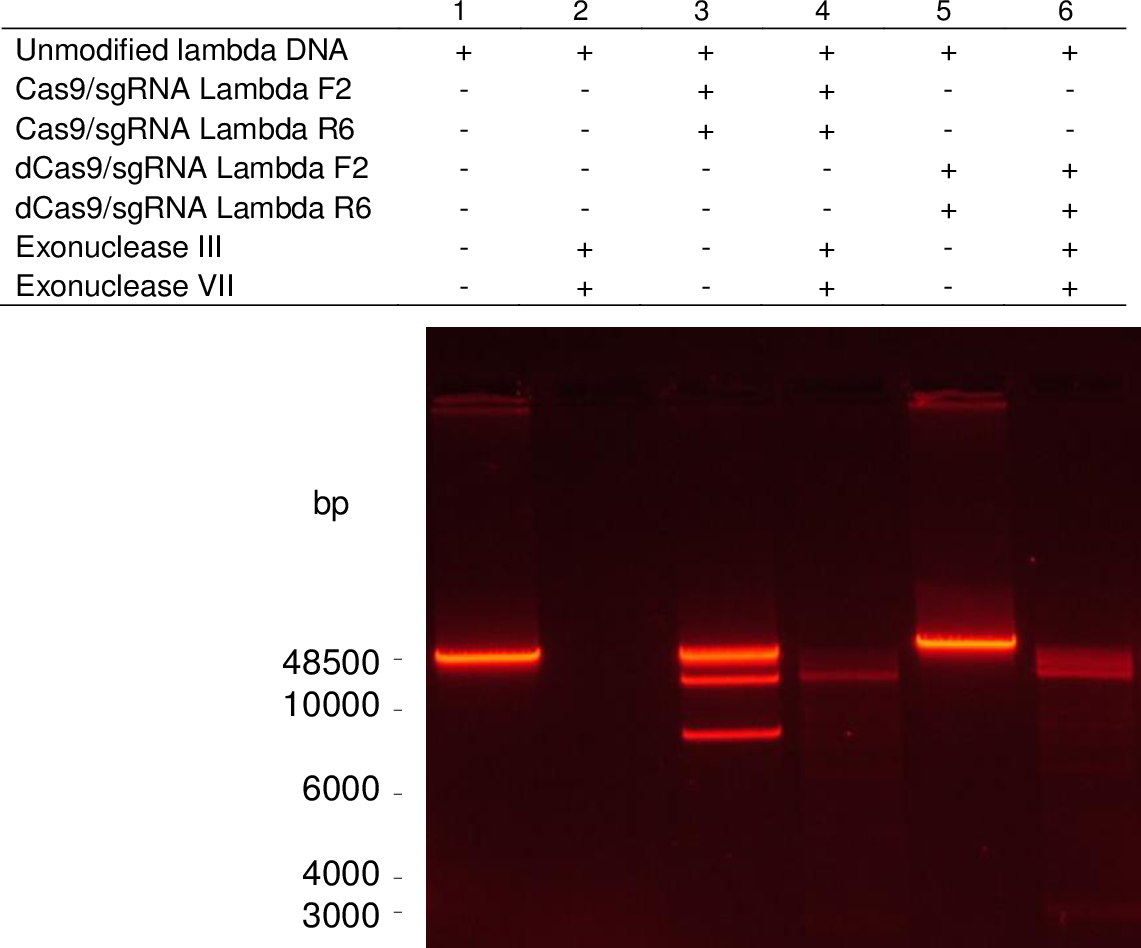

Supplement: S1 Fig — Gel electrophoresis of lambda DNA without or with exonuclease III and exonuclease VII treatment (lanes 1 and 2); lambda DNA with Cas9 complexed to Lambda F2 and Lambda R6 without or with exonuclease III and exonuclease VII (lane 3 and 4); lambda DNA with dCas9 complexed to Lambda F2 and Lambda R6 without or with exonuclease III and exonuclease VII (lane 5 and 6). (TIF) [file pone.0215441.s004.tif]

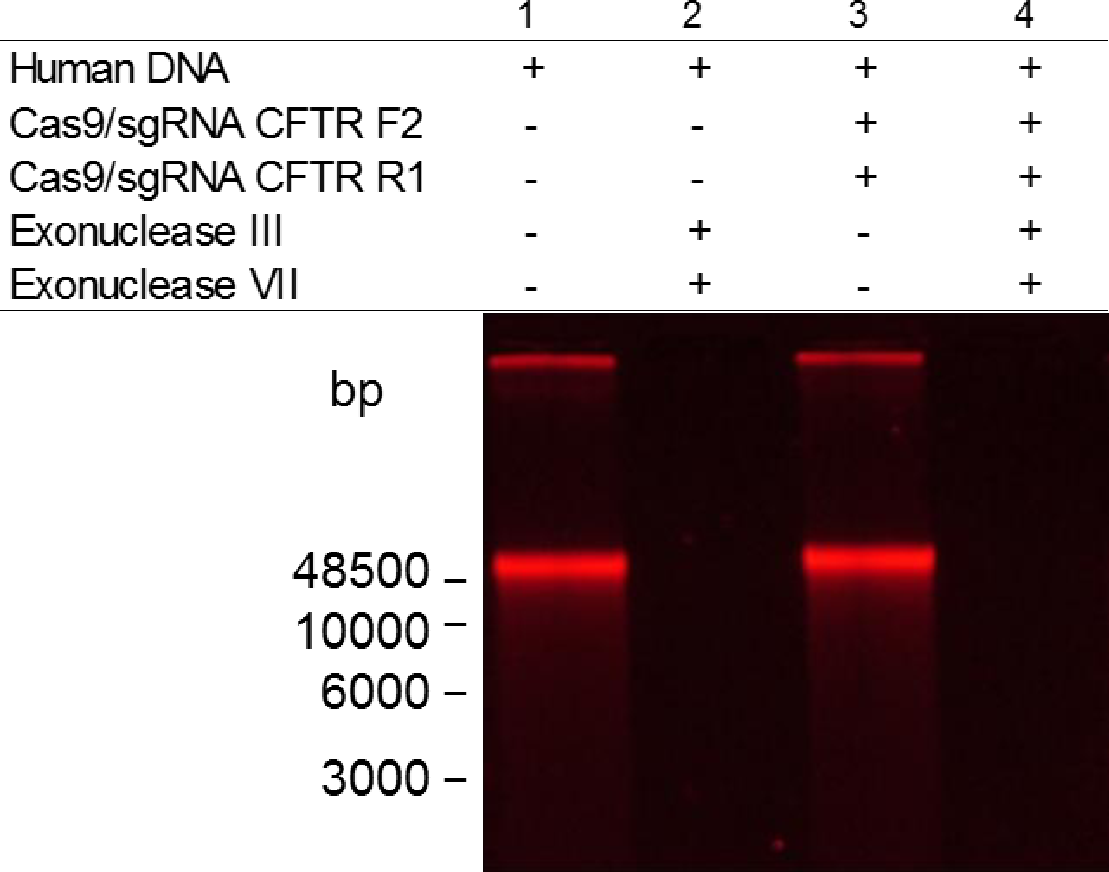

Supplement: S2 Fig — Gel electrophoresis showing human genomic DNA without or with exonuclease treatment (lanes 1 and 2), and human genomic DNA complexed with Cas9/sgRNAs CFTR F2 and CFTR R1 without or with exonuclease treatment (lanes 3 and 4). (TIF) [file pone.0215441.s005.tif]

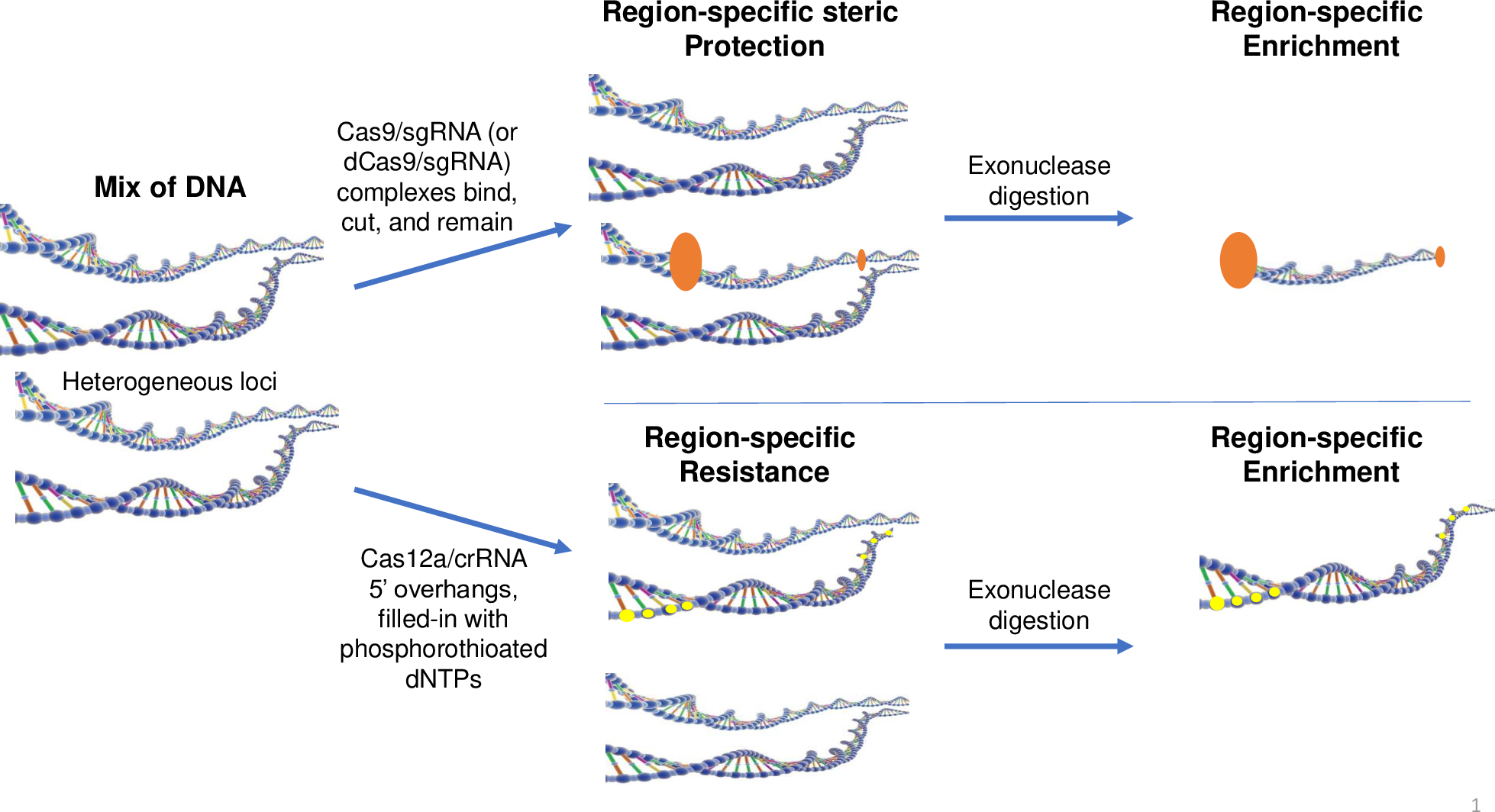

Supplement: S3 Fig — (TIF) [file pone.0215441.s006.tif]

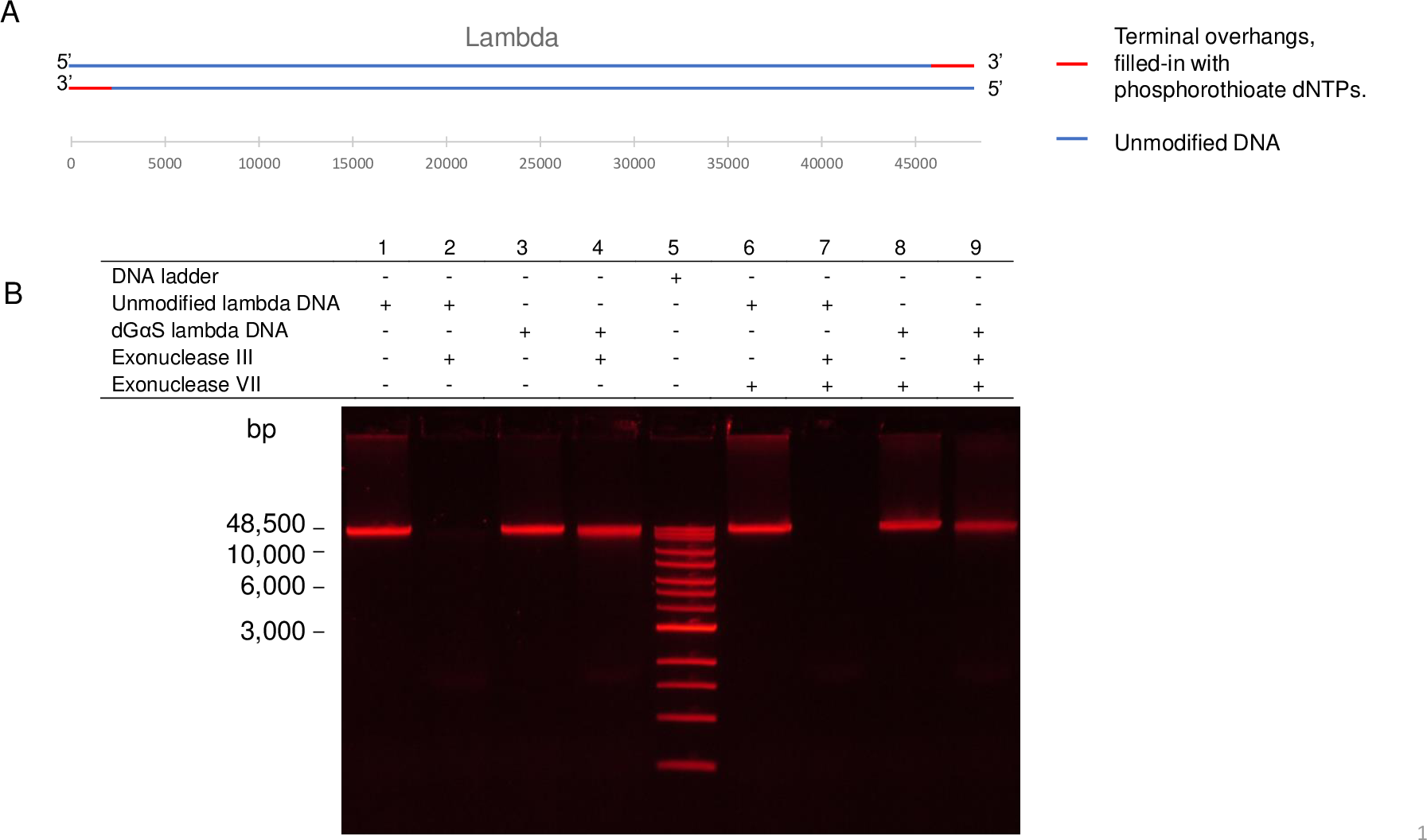

Supplement: S4 Fig — (A) Diagram showing intact lambda genomic DNA cos sites modified via polymerase incorporation of dGTP-αS modifications. (B) A 0.7% agarose gel shows lambda DNA filled in with wild-type dNTPs (lane 1), lambda DNA filled in with wild-type dNTPs and incubated with exonuclease III (lane 2), lambda DNA filled in with phosphorothioated bases (dGαS lambda DNA) (lane 3), dGαS lambda DNA incubated with exonuclease III (lane 4), lanes 6–9 duplicate the conditions of 1–4 with the addition of exonuclease VII. (TIF) [file pone.0215441.s007.tif]

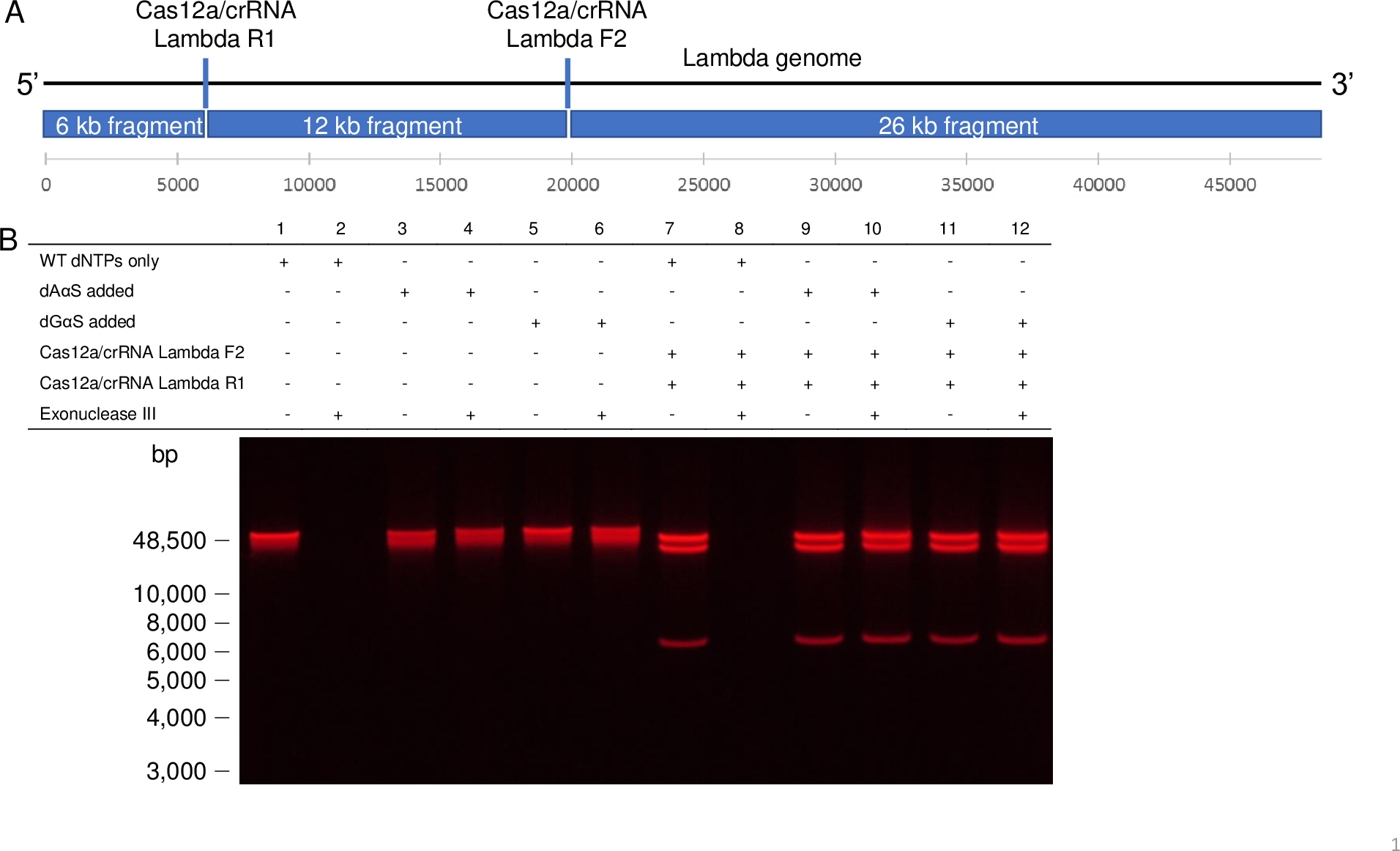

Supplement: S5 Fig — (A) Diagram of lambda genomic DNA shows the relative positions of complexes Cas12a/crRNA Lambda R1 and Cas12a-crRNA Lambda F2: (B) A 0.7% agarose gel shows lambda DNA filled in with wild-type dNTPs (lane 1), lambda DNA filled in with wild-type dNTPs and incubated with exonuclease III (lane 2), lambda DNA filled in with phosphorothioated bases (dAαS lambda DNA) (lane 3), dAαS lambda DNA incubated with exonuclease III (lane 4), lambda DNA filled in with phosphorothioated bases (dGαS lambda DNA) (lane 5), dGαS lambda DNA incubated with exonuclease III (lane 6). Lambda DNA treated with Cas12a/crRNA and filled in with wild-type dNTPs (lane 7), lambda DNA filled in with wild-type dNTPs and incubated with exonuclease III (lane 8), lambda DNA filled in with phosphorothioated bases (dAαS lambda DNA) (lane 9), dAαS lambda DNA incubated with exonuclease III (lane 10), lambda DNA filled in with phosphorothioated bases (dGαS lambda DNA) (lane 11), dGαS lambda DNA incubated with exonuclease III (lane 12). (TIF) [file pone.0215441.s008.tif]
