# Supplementary material for: A novel CRISPR/Cas9 associated technology for sequence-specific nucleic acid enrichment
Source: PLoS One. 2019 Apr 18;14(4):e0215441. doi: 10.1371/journal.pone.0215441 (PMC6472885; doi:10.1371/journal.pone.0215441)
Supplement: S1 File — (DOCX) [file pone.0215441.s009.docx]

## Alternative Negative Enrichment using phosphorothioated nucleotides and Cas12a

**Introduction**

## In addition to using Cas9 for Negative Enrichment, a different Cas variant, Cas12a, was used which leaves a 4-8 base overhang after cleaving the target site. Polymerases were then used to add phosphorothioated nucleotides to create blunt ends resistant to exonuclease digestion (S3 Fig).

**Methods**

The use of phosphorothioated nucleotides to protect bacteriophage lambda DNA from exonuclease digestion was first examined by utilizing Klenow enzyme (NEB, cat. #M0210L) to fill in the12 base cos-site overhangs of lambda DNA with a mixture of three unmodified and one phosphorothioated nucleotide triphosphates (dTTP, dCTP, dATP and Sp-dGTPαS, NEB, cat. #N0446S and Axxora, cat. #BLG-D031-05) to generate dGαS lambda DNA. Samples were purified using BioRad Kleen columns for further use. Samples were then treated with exonuclease III (NEB, cat. #M0206L) and exonuclease VII (NEB, cat. #M0379L) and analyzed by gel electrophoresis.

Protection using Cas12a was completed with the addition of two crRNAs shown in S1 Table. After the formation of the Cas12a/crRNA complexes, 330 ng of lambda DNA was added. The reactions were ended with the addition of proteinase K (NEB, cat. #P8107S) and incubation at 56°C. Samples were purified using BioRad Kleen columns and the cos site overhangs were filled in using the methods previously described (see above). Finally, samples were treated with exonuclease III and evaluated by gel electrophoresis.

**Results**

Protection of full-length lambda DNA after filling in the 12-base cos sites with phosphorothioated dGTPαS is shown in S4 Fig. Lanes 1-4 show digestion with exonuclease III alone and lanes 6-9 show digestion with exonuclease III and VII. In both cases, lambda DNA that has been modified with dGTPαS remains after exonuclease digestion (lanes 4 and 9) while unmodified lambda DNA is degraded (lane 2 and 7).

Protection of Cas12a cleaved lambda DNA is shown in S5 Fig. In this figure, lanes 1-6 replicate the results of S4 Fig. The remaining lanes show the results of cleavage with Cas12a followed by extension with unmodified (lanes 7 and 8) and phosphorothioated (lanes 9-12) nucleotides. After the extension reaction, protection was demonstrated by treatment with exonuclease III (lanes 8, 10 and 12).

**Discussion**

In addition to the Negative Enrichment approach demonstrated in the main text of this manuscript using Cas9, this technique can be extended to a wide variety of CRISPR enzymes. Here we have verified this with the use of Cas12a and phosphorothioated nucleotides in protection of lambda DNA.
