# Supplementary material for: A novel CRISPR/Cas9 associated technology for sequence-specific nucleic acid enrichment
Source: PLoS One. 2019 Apr 18;14(4):e0215441. doi: 10.1371/journal.pone.0215441 (PMC6472885; doi:10.1371/journal.pone.0215441)
Supplement: S2 File — (DOCX) [file pone.0215441.s010.docx]

**Cost comparisons for Negative Enrichment and whole genome sequencing**

One of the purposes of DNA sample enrichment is to reduce the amount of total sequencing required in order to adequately sequence a targeted locus. This reduction of total sequencing has a direct cost benefit, as illustrated in S3 Table.

In this analysis, we compare the cost of sequencing two samples: an unenriched control and a Negatively Enrichment sample. We calculated the total amount of sequence data that would provide a coverage level of 2 or greater for 99.99% of the positions within the targeted locus (reads required). The total number of required reads is substantially lower for the enriched sample. The enriched sample takes an additional half day of preparation; this is more than recovered by the lower amount of DNA needed in subsequent QC steps and the proportion of an Illumina sequencing run. Overall, with a 24x enrichment ratio, this example results in 93% cost reduction per sample.
